# Radiology as a career among medical students of Pakistan: A cross-sectional study

**DOI:** 10.1097/MD.0000000000038156

**Published:** 2024-05-17

**Authors:** Muhammad Junaid Tahir, Hashaam Jamil, Razia Jabbar, Mohsin Khalid Qureshi, Muhammad Hamayl Zeeshan, Irfan Ullah, Abubakar Nazir, Muna Malik, Mohammed Mahmmoud Fadelallah Eljack, Muhammad Sohaib Asghar

**Affiliations:** a Department of Radiology, Pakistan Kidney and Liver Institute and Research Center, Lahore, Pakistan; b Department of Medicine, Pakistan Kidney and Liver Institute and Research Center, Lahore, Pakistan; c Department of Medicine, Lahore General Hospital, Lahore, Pakistan; d Department of Medicine, HBS Medical and Dental College, Islamabad, Pakistan; e Department of Medicine, Dow University of Health Sciences, Karachi, Pakistan; f Department of Medicine, Kabir Medical College, Gandhara University, Peshawar, Pakistan; g Department of Medicine, King Edward Medical University, Lahore, Pakistan; h Department of Pathology, King Edward Medical University, Lahore, Pakistan; i Department of Medicine, Faculty of Medicine and Health Sciences, University of Bakhtalruda, Al-Dewaym, Sudan; j Department of Medicine, Mayo Clinic, Rochester, MN.

**Keywords:** career, diagnosis, education, imaging, medical graduates, Radiology

## Abstract

Radiology has become a fundamental constituent of the modern medicine. However, it has been observed that medical students in Pakistan often lack sufficient guidance and education in this field. This study aims to establish whether Pakistani medical students possess the requisite basic knowledge required in radiology and their attitude and perception toward radiology as a potential career path. This cross-sectional study conducted a survey among 530 medical students of Pakistan via a self-reported online questionnaire from August 01, 2021 to September 01, 2021. The data collected were analyzed using the SPSS software, along with logistic regression analyses to identify factors associated with interest in pursuing radiology as a career and possessing a comprehensive understanding of radiology among medical students. Of the 530 participants, 44.2% rated their understanding of radiology as “poor” with only 17% indicating interest to pursue a career in radiology. Logistic regression model showed significantly higher odds of radiology as a career among males (Crude odds ratio [COR] = 1.78, 95% confidence interval [CI] = 1.17–2.72, *P* = .007), medical students of Punjab (COR = 1.55, 95% CI = 1.01–2.40, *P* = .048), and those, who self-reported their knowledge of radiology as excellent (COR = 14.35, 95% CI = 5.13–40.12, *P* < .001). In contrast, medical students from Punjab (COR = 0.504, 95% CI = 0.344–0.737, *P* < .001) and second-year medical students (COR = 0.046, 95% CI = 0.019–0.107, *P* < .001) had lower odds of good knowledge. Our study suggests that the medical student’s knowledge of radiology is deficient. Thus, it is advised that radiological societies work with medical school boards to integrate thorough and early radiology exposure into the undergraduate curriculum.

**Key Points**

1. This study aimed to determine the knowledge and attitudes of Pakistani medical students toward radiology as a career.
2. About 44.2% of participants rated their understanding of radiology as poor, and only 17% showed interest in pursuing a career in radiology.
3. Logistic regression showed higher odds of pursuing radiology as a career for male students, and those who self-reported their knowledge of radiology as excellent.
4. The study highlights a deficiency in radiology knowledge among medical students in Pakistan and recommends integrating comprehensive radiology exposure into the undergraduate curriculum through collaboration between radiological societies and medical school boards.

## 1. Introduction

Radiology is a specialty of medicine in which images of the body’s organs are interpreted in order to diagnose diseases crucial for nearly every sector of healthcare, including surgery, pediatrics, obstetrics, cancer care, trauma response, emergency medicine, infectious disease, and much more.^[1]^ Radiology has become more important as contemporary medicine has evolved. It has the ability to efficiently diagnose and reduce unnecessary procedures safely and effectively. With the advancement in radiological modalities, healthcare practitioners are increasingly requiring medical imaging.^[2,3]^ Despite its significance, it has been observed that medical students are not receiving adequate education in radiology.^[4,5]^ In most traditional medical school curricula, radiology is not explicitly introduced to the students until their clinical rotations; even then, radiology is not always included in the basic curriculum.^[6]^ Radiology is only introduced as a minor component of anatomy or organ pathology courses during the preclinical years, although dedicated lectures are uncommon.^[7]^

Multiple studies have shown that medical students worldwide lack the fundamental knowledge required for imaging indications and interpretations.^[8–10]^ In Pakistan, the majority of medical students have limited knowledge about radiology, especially in their preclinical years.^[11]^ Furthermore, medical students in Pakistan are not well versed when it comes to dosage and hazards that come with the utilization of radiological imaging.^[12]^ In 2018, a study was conducted on the specialty preferences of Pakistani medical students, which revealed only 9% of medical students expressed interest in becoming radiologists in the near future.^[13]^ However, it is surprising to know how little is being done in Pakistan to promote radiology. In Pakistan, medical students attend their clinical rotations in the department of radiology either in the fourth or final year of their undergraduate training. Within the curriculum, the Pakistan Medical and Dental Council has combined 6 subjects that include radiology and has allocated a total of 10 to 12 lectures in 5 years.^[14]^ Early exposure to radiology education in the undergraduate medical curriculum provides medical students with the ability to interpret basic radiographs, understand human anatomy, and develop a greater interest in radiology as a career.^[15–17]^ Because of late exposure to radiology, medical students do not exhibit any interest in radiology until late in their training,^[18]^ and more likely to harbor negative stereotypes about radiologists.^[19,20]^ Therefore, students should have sufficient knowledge about radiology, as it is a vital part of their future medical practice. This study aims to ascertain whether Pakistani medical students possess the fundamental knowledge required in radiology. A secondary aim is to assess their attitude toward radiology as a specialty and to determine the factors associated with good knowledge,

## 2. Methodology

### 2.1. Study design and setting

A cross-sectional survey was carried out among medical students in Pakistan, employing a convenience sampling technique. A semistructured questionnaire including informed consent was incorporated into the Google Forms and a shareable link was created. Survey responses were collected between August 01, 2021 to September 01, 2021, in the 4 provinces of Pakistan, (i.e., Sindh, Punjab, Balochistan, and Khyber Pakhtunkhwa).

### 2.2. Sample size

The sample size was calculated by using online sample size calculator software (i.e., Raosoft, Seattle) with a 95% confidence interval (CI), 50% as the online survey response rate, and a 5% margin of error. Hence, the minimum number of samples needed for this study was calculated as 377. To obtain more reliable and precise results, we eventually received 530 responses to include in the study. Incomplete and duplicate submissions of the survey questionnaire were not possible due to the feature in Google Forms that prevented the submission of partially filled questions and duplication of responses.

### 2.3. Questionnaire development

The questionnaire was developed through an intensive review of the literature,^[10,17,21]^ and reviewed by the research committee comprised of senior radiologists and other medical professionals with relevant research expertise. After discussion and review, the authors finalized the questionnaire. A pilot study was carried out with a small group of medical students to determine its reliability before the questionnaire was used in the study. The data from these participants were not included in the final analysis. A minimum Cronbach Alpha of 0.7 was considered acceptable reliability.^[22]^ Finally, the questionnaire was distributed among the participants to collect their responses. The questionnaire comprised an introductory paragraph, clarifying the aim and objectives of the study; followed by mandatory informed consent for all participants; and then 3 sections assessing demographics, knowledge, and attitude.

The general characteristic section consisted of 9 questions, 5 of which were demographics in nature (i.e., age, gender, residence, medical university, and year of medical school). The knowledge section consisted of 10 questions. The correct answer was coded as 1, while the wrong answer was coded as 0. The total score was obtained by summing the raw score of each item and ranged from 0 to 10, with an overall higher score indicating more precise knowledge. And the last section consisted of 5 questions to assess the attitude of medical students toward radiology.

### 2.4. Data collection and sampling

All medical undergraduates of Pakistan from the first year to final year of MBBS, aged ≥18 years of either gender (male or female), were eligible to participate in the survey. Those participants who refused to give their informed consent were excluded from the study. The questionnaire was designed in English. Data were collected through friend circle forwarding, WhatsApp sharing, and other social media platforms (e.g., Facebook, Gmail). The survey was completely voluntary, and participants could withdraw their responses from the survey at any moment, as per their choice.

### 2.5. Ethical considerations

Students were informed that their participation is voluntary and their anonymity will be assured. The study did not include any names or emails, so the participants could not be tracked. Participants were allowed to withhold the completed form at any point before submitting it. The study protocol was approved by the Ethical Review Committee of a medical teaching institution, Lahore General Hospital, Lahore, Pakistan (Ref. No: 00/49/21).

### 2.6. Statistical analysis

The data obtained from the structured questionnaire were entered into a computer to generate a computerized database for subsequent analysis using the Statistical Package for the Social Sciences (SPSS, Chicago) version 21. Descriptive statistics were applied as means and standard deviations for continuous variables, while categorical variables were expressed using frequencies and percentages. Using univariate and multivariate logistic regression models, we assessed potential contributing factors for radiology as a career and good knowledge of radiology. A *P* value of < 0.05 was considered statistically significant.

## 3. Results

### 3.1. General characteristics and demographics

**Figure 1.**
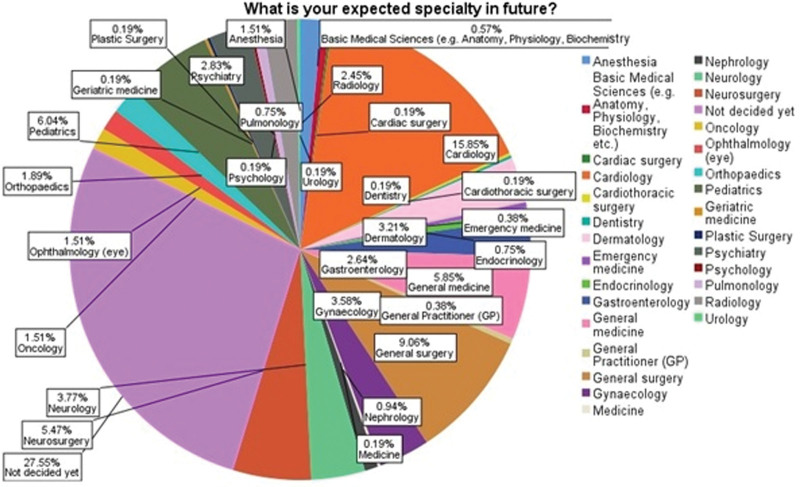
Career choice among medical students.

A total of 530 participants with a mean age of 21.18 ± 1.75 were included in the final analysis. Of them, the majority were male medical students (66.0%), residents of Punjab (66.0%), from public medical universities (91.9%), and studying in their third year (26.2%). When asked about career choice in the future, 27.5% had not decided yet, and a few students (2.4%) selected radiology as a career. (Fig. 1) Among participants, 44.2% self-reported that they had poor knowledge of radiology (Table 1).

**Table 1:** General characteristics and demographics of the study participants (N = 530).

Variables	Characteristics	Descriptive/frequency
Age (yr)	Mean (SD)	21.18 (1.75)
Gender	Female	180 (34.0%)
	Male	350 (66.0%)
State (residence)	Sindh	161 (30.4%)
	Punjab	350 (66.0%)
	Others	19 (3.6%)
Medical university	Private	43 (8.1%)
	Public	487 (91.9%)
Year of medical school	First	89 (16.8%)
	Second	106 (20.0%)
	Third	139 (26.2%)
	Fourth	125 (23.6%)
	Final	71 (13.4%)
Career choice	Clinical	509 (96.0%)
	Nonclinical (basic sciences)	21 (4.0%)
If you have planned for radiology as a career in future, which specialization you would choose?	Diagnostic radiology	94 (17.7%)
	Interventional radiology	60 (11.3%)
	Any one of the above 2	72 (13.6%)
	Not applicable	304 (57.4%)
In your academic years (till now), how many weeks you have done rotations and/or electives in radiology?	Mean (SD)	3.70 (4.19)
How would you rate your knowledge of radiology as compared to other fields?	Excellent	25 (4.7%)
	Good	81 (15.3%)
	Fair	190 (35.8%)
	Poor	234 (44.2%)

SD = standard deviation.

### 3.2. General knowledge of radiology

Table 2 presents the knowledge of medical students regarding radiology. Most participants were aware that children are the most sensitive to the radiations (58.5%), ultrasound is the safest imaging modality (61.1%), and also that the radiations affect the fetus (92.3%). There was a misunderstanding about which imaging modality is the most hazardous to the fetus as 37.2% of students thought it was an X-ray, while only 26.8% of students correctly recognized it as computed tomography (CT) imaging. Regarding contraindications, 52.4% believed that pregnancy is a contraindication to CT scan and 50.7% believed that metal foreign body is a contraindication to magnetic resonance imaging. Among participants, 55.8% were aware that mammography is used for breast cancer screening. The knowledge scores were high in residents of Sindh (*P* < .001), higher undergraduate levels (*P* < .001), career choice as clinical sciences (*P* = .038), aspiring interventional radiologist (*P* = .015), and with self-rated knowledge of radiology (*P* = .022), as shown in Table 3.

**Table 2:** Knowledge questions regarding radiology and responses of the study participants (N = 530).

Variables	Characteristics	Frequency (%)
Which age group is the most sensitive to radiation?	**Children (0–12 yr**)	310 (58.5%)
	Teens (13–18 yr)	29 (5.5%)
	Adults (19–50 yr)	34 (6.4%)
	Elders (above 50 yr)	59 (11.1%)
	Do not know	98 (18.5%)
What is the safest imaging technique?	X-ray	44 (8.3%)
	**Ultrasound**	324 (61.1%)
	CT scan	19 (3.6%)
	MRI	64 (12.1%)
	Do not know	79 (14.9%)
What are the most sensitive organs to radiation?	Brain	95 (17.9%)
	Breast	34 (6.4%)
	Liver, bladder, kidney	67 (12.6%)
	Lungs, colon	19 (3.6%)
	**Testis and ovaries**	181 (34.2%)
	Do not know	134 (25.3%)
Is there a specific number of radio graphs that can be requested for the patient per year?	Yes	249 (47.0%)
	**No**	54 (10.2%)
	Do not know	227 (42.8%)
Does radiation affect the fetus?	**Yes**	489 (92.3%)
	No	8 (1.5%)
	Do not know	33 (6.2%)
What type of imaging technique most affects the fetus?	X-ray	197 (37.2%)
	Ultrasound	44 (8.3%)
	**CT scan**	142 (26.8%)
	MRI	50 (9.4%)
	Do not know	97 (18.3%)
Conventional CT scan equivalent to how many chest X-ray?	10–100	76 (14.3%)
	**100–500**	80 (15.1%)
	500–1000	58 (10.9%)
	1000–1500	28 (5.3%)
	Do not know	288 (54.3%)
Contraindications of CT scan (select all that apply)	**Allergy to radio-contrast agent**	240 (45.3%)
	**Renal failure**	128 (24.1%)
	**Liver failure**	74 (14.0%)
	**Pregnant women**	278 (52.4%)
	Do not know	171 (32.3%)
Contraindications of MRI (select all that apply)	**Pacemaker**	226 (42.6%)
	**Metal foreign bodies**	269 (50.7%)
	**Claustrophobia (irrational fear of confined or small space**)	222 (41.9%)
	Don’t know	179 (33.8%)
Radiology use as screening test in (select all that apply)	**Mammography for breast cancer**	296 (55.8%)
	**Ultrasound for abdominal aortic aneurysm**	198 (37.4%)
	**CT for lung cancer**	198 (37.4%)
	**DEXA for osteoporosis**	154 (29.1%)
	Do not know	162 (30.6%)
Source(s) of information on radiology for you? (select all that apply)	Internet	312 (58.9%)
	Social media	143 (27.0%)
	Lectures	208 (39.2%)
	Personal experience	143 (27.0%)
	Clinical rotations	108 (20.4%)
	Friends	94 (17.7%)
	Radiology research experience	30 (5.7%)
	Radiologist family member	32 (6.0%)
	Attended a radiology conference	28 (5.3%)

Categories in bold represent correct answers (each carry 1 score to formulate a total of 10 points).

CT = computerized tomography, MRI = magnetic resonance imaging.

**Table 3:** Scores of knowledge with comparison among the independent variables (N = 530).

Variables	Characteristics	Mean (SD) knowledge scores	*P* value
Gender	Female	4.99 (2.19)	.224
	Male	5.23 (2.09)	
State (residence)	Sindh	5.80 (1.66)	<.001
	Punjab	4.77 (2.31)	
	Others	4.63 (1.73)	
Medical university	Private	5.02 (1.94)	.860
	Public	5.08 (2.18)	
Year of medical school	First	4.16 (2.23)	<.001
	Second	4.13 (2.13)	
	Third	4.71 (2.12)	
	Fourth	5.81 (1.52)	
	Final	7.04 (1.32)	
Career choice	Clinical	5.12 (2.14)	.038
	Nonclinical (basic sciences)	3.95 (2.39)	
If you have planned for Radiology as a career in future, which specialization you would choose?	Diagnostic radiology	5.28 (1.91)	.015
	Interventional radiology	6.30 (1.77)	
	Any one of the above 2	4.75 (2.23)	
	Not applicable	4.85 (2.21)	
How would you rate your knowledge of radiology as compared to other fields?	Excellent	5.36 (1.84)	.022
	Good	5.32 (2.07)	
	Fair	5.34 (2.14)	
	Poor	4.75 (2.21)	

SD = standard deviation.

### 3.3. Attitude toward radiology as a specialty

More than half of the students (58.1%) considered they had barely been introduced to radiology, and just a few students (2.5%) believed that they knew more about radiology than any other specialty. In terms of radiology exposure in medical school, some students (30.2%) reported that they have not been exposed to radiology and some (27.5%) reported that they have encountered radiology as a minor part of another course. About half of the participants (47.7%) thought that radiology is as important as a physical examination. About 28.5% were considering radiology as a career in the future (Table 4).

**Table 4:** Attitude of the study participants toward radiology as a specialty (N = 530).

Variables	Characteristics	Frequency (%)
How much do you know about the radiology as a specialty?	I know more about radiology than any other specialty	13 (2.5%)
	I am about as familiar with radiology as with any other specialty	175 (33.0%)
	I have barely been introduced to radiology	308 (58.1%)
	I’ve never heard of radiology	38 (6.4%)
How much radiology have you been exposed to in medical school?	None	160 (30.2%)
	1 or 2 dedicated lectures	72 (13.6%)
	Only in passing	125 (23.6%)
	Peripherally, as a minor part of another course	146 (27.5%)
	Several lectures and study sessions	27 (5.1%)
How interesting is the subject matter in radiology?	It is worthless to me	27 (5.1%)
	It is dull but important	127 (24.0%)
	Peripherally, as a minor part of another course	105 (19.8%)
	It is interesting in its own right	236 (44.5%)
	It is fascinating	35 (6.6%)
How much of an impact does radiology have on other areas of medicine?	Minimal impact	38 (7.2%)
	Occasionally changes patient care	37 (7.0%)
	Just as important as physical examination	253 (47.7%)
	More important than physical examination	97 (18.3%)
	Often changes patient care	105 (19.8%)
Are you considering radiology as a career in future?	No	379 (71.5%)
	Yes	151 (28.5%)

Data presented as frequency and percentages.

### 3.4. Factors associated with radiology as a career and good knowledge of radiology

Univariate regression analysis showed significantly higher odds of radiology as a career among males (Crude odds ratio [COR] = 1.78, 95% CI = 1.17–2.72, *P* = .007) compared with females. Medical students of the private medical university (COR = 1.92, 95% CI = 1.01–3.63, *P* = .046), Punjab (COR = 1.55, 95% CI = 1.01–2.40, *P* = .048), and those, who self-reported their knowledge of radiology as excellent (COR = 14.35, 95% CI = 5.13–40.12, *P* < .001) had a statistically significant association with willingness to choose radiology as a career (Table 5).

**Table 5:** Regression analysis of factors associated with choosing radiology as future career choice.

Variables	Univariate	*P* value	Multivariate	*P* value
Gender				
Female	1.000	-	1.000	-
Male	1.782 (1.168–2.718)	.007*	1.498 (0.945–2.374)	.086
Medical university				
Public	1.000	-	1.000	-
Private	1.916 (1.013–3.626)	.046*	1.602 (0.801–3.204)	.183
State (residence)				
Sindh	1.000	-	1.000	-
Punjab	1.550 (1.003–2.393)	.048*	1.069 (0.663–1.724)	.783
Others	2.025 (0.743–5.523)	.168	0.989 (0.320–3.057)	.984
Year of medical school				
First	0.845 (0.436–1.636)	.617	-	-
Second	0.629 (0.327–1.210)	.165	-	-
Third	0.797 (0.434–1.462)	.463	-	-
Fourth	0.556 (0.293–1.054)	.072	-	-
Fifth	1.000		-	-
How would you rate your knowledge of Radiology as compared to other fields?				
Excellent	14.353 (5.135–40.121)	<.001*	12.377 (4.328–35.398)	<.001*
Good	2.467 (1.436–4.238)	.001*	2.210 (1.269–3.847)	.005*
Fair	1.179 (0.750–1.855)	.475	1.139 (0.720–1.802)	.579
Poor	1.000	-	1.000	-
Career choice				
Clinical	0.635 (0.258–1.564)	.323	-	-
Nonclinical (basic sciences)	1.000	-	-	-

* Significant *P* values.

Medical students from Punjab had low odds of knowledge compared with the medical students of Sindh (COR = 0.504, 95% CI = 0.344–0.737, *P* < .001; adjusted odds ratio [AOR] = 0.563, 95% CI = 0.340–0.933, *P* = .026). An increase in the medical school year is associated with good knowledge, as first-year and second-year medical students had lower odds of knowledge, compared with final-year medical students (COR = 0.065, 95% CI = 0.027–0.152, *P* < .001; AOR = 0.058, 95% CI = 0.024–0.139, *P* < .001) and (COR = 0.046, 95% CI = 0.019–0.107, *P* < .001; AOR = 0.046, 95% CI = 0.019–0.109, *P* < .001) respectively (Table 6).

**Table 6:** Regression analysis of factors associated with good knowledge of radiology questions (cutoff score ≥6).

Variables	Mean knowledge scores†	Univariate	*P* value	Multivariate	*P* value
Gender					
Female	5.23 (2.09)	1.253 (0.874–1.796)	.219	0.704 (0.455–1.090)	.115
Male	4.99 (2.19)	1.000	-	1.000	-
Medical university					
Public	5.08 (2.18)	1.000	-	1.000	-
Private	5.02 (1.94)	0.895 (0.479–1.672)	.728	0.878 (0.429–1.799)	.723
State (residence)					
Sindh	5.80 (1.66)	1.000	-	1.000	-
Punjab	4.77 (2.30)	0.504 (0.344–0.737)	<.001*	0.563 (0.340–0.933)	.026*
Others	4.63 (1.73)	0.224 (0.077–0.652)	.006*	0.354 (0.107–1.172)	.089
Year of medical school					
First	4.16 (2.23)	0.065 (0.027–0.152)	<.001*	0.058 (0.024–0.139)	<.001*
Second	4.13 (2.13)	0.046 (0.019–0.107)	<.001*	0.046 (0.019–0.109)	<.001*
Third	4.71 (2.12)	0.108 (0.048–0.243)	<.001*	0.100 (0.044–0.227)	<.001*
Fourth	5.81 (1.52)	0.190 (0.084–0.432)	<.001*	0.138 (0.058–0.331)	<.001*
Fifth	7.04 (1.32)	1.000		1.000	-
Career choice					
Clinical	5.12 (2.14)	2.490 (0.951–6.520)	.063	2.546 (0.890–7.281)	.081
Nonclinical (basic sciences)	3.95 (2.39)	1.000	-	1.000	-

Overall mean knowledge scores = 5.08 (2.16).

* Significant *P* values.

† Data presented as mean (standard deviation).

## 4. Discussion

The purpose of this study was to conduct a comprehensive survey among Pakistani medical students regarding their overall knowledge and possible interest in radiology. Many determinants influence a student’s attitude toward any specialty and it is pertinent to identify and rule out those factors that discourage students from pursuing radiology. By identifying the factors that foster an inclination toward radiology, it becomes possible to take relevant measures to emphasize and augment these aspects, thereby enhancing students’ interest in this field.

In our study, it was reported that women were less likely to choose radiology as a career option compared with their male counterparts. Globally, even though women make up nearly half of the classes in their medical schools, only 22% represent practicing radiologists overall.^[23]^ Previous literature has shown that female participants are less likely to choose radiology as a career compared with males,^[23–26]^ this is consistent with our study. In Pakistan, the female lifestyle consists of great attention to the upbringing of their child, with considerable concern for their family overall. These factors could be a cause for this disparity.

Regardless, there has been a rise in the trend of females opting for radiology recently. According to the 2015 American College of Radiology workforce survey, around 31% of practicing radiologists younger than 35 years of age were women.^[27]^ Factors such as an increase in awareness and efforts by national organizations such as the American Association for Women Radiologists help promote this trend.^[28]^ Another factor that proves to be quite beneficial is the encouragement of mentorship programs. To support this hypothesis, a study was conducted at the University of Michigan, where the first-year medical students were surveyed before and after a 7-week radiology course, and their responses were recorded. For women, one of the most common factors for choosing radiology that stood out was the influence of the mentor; they believe that having a mentor influences their decision.^[29]^ This suggests that radiology chairs should make mentorship programs for medical students a priority.

In our study, 61.1% of students successfully identified ultrasound as the safest imaging method. Surprisingly, a considerable number of students incorrectly mentioned that X-ray is the most hazardous to the fetus compared to ultrasound, which identified a knowledge gap regarding imaging-related risks among Pakistani medical students. Regarding contraindications, even though the majority correctly identified it for both, CT (52.4%) and magnetic resonance imaging (50.7%), at the same time, a significant number of participants could not. This creates a question about the knowledge gap about imaging contraindications as well. A study that supports this finding by Alchallah et al^[21]^ indicates that only 8.2% of the total participants correctly identified all the contraindications and the rest only identified just a couple for both imaging modalities. Another study by Prezzia et al^[19]^ also supports this finding where it was observed that only 26.4% of the total participants were able to identify all of the correct answers.

In many schools, radiologists have very little teaching role in the medical curriculum, and because of this, students are unable to see radiologists in complete action compared to other specialties. To improve this situation, radiologists can look for opportunities that may help expand their role in the teaching department and as a result, can improve the image of radiologists among medical students.^[6]^ Radiology also helps in other medical subjects. A study by Chew et al^[30]^ demonstrated that studying radiology had a significant improvement in anatomy scores among medical students.

Our study also demonstrated that 44.2% of students considered they had “‘poor’” knowledge of radiology and only a few students (2.4%) wanted to pursue a career in radiology. One of the reasons for these numbers is because of the lack of exposure to radiology in medical school. Literature suggests that medical students who were exposed to radiology during their medical school were more influenced in choosing radiology in the future.^[25]^

To create interest in a career in radiology, it is important to introduce radiology rotation from the first year of medical school. Different studies have shown that the majority of medical schools do not emphasize radiology during the first year of medical school. Alchallah et al^[21]^ observed that 73% of students did not complete their radiology rotation in their first year. Alnajjar et al^[31]^ identified only 25% of Saudi medical students completed their rotation and Agrawal et al^[32]^ observed only 5.7% of Indian medical students in their study.

According to our study, only 2.5% of students mentioned that they knew radiology more compared to other specialties. The research gap for choosing radiology among other specialties still needs exploring. Regardless of specialty, a good grip on radiology can be a useful asset for any medical practitioner. Medical imaging is one of the most important diagnostic tools available as it is utilized by every medical specialty. Usually, medical students are taught that history and examination are the main criteria to make a diagnosis and radiology does not play a significant role, but our study showed that the majority of students (47%) termed radiology to be as important as the other 2. Early introduction to radiology may prove to be beneficial but the clinical experience during their third, fourth, and final year rotations would have a greater impact in terms of realizing its significance.

A way to approach this is to introduce more active learning for radiology. When compared to other specialties such as general surgery and internal medicine where the learning experience is more hands-on, radiology rotations tend to be more passive than active; resulting in a less stimulating and satisfying experience for medical students. To add to this, it was demonstrated in a study that students who were actively taught radiology had a positive perception of the field.^[33]^ They were more comfortable interacting with the radiology department upon graduation and were more open to the idea of choosing radiology in the future. Other literature studies have also demonstrated an improvement in the student’s attitude toward radiology after positive radiologist-delivered education.^[6,34]^ This gives us the idea that promoting education via active learning and practical methods can induce a positive response from the students.

Despite this, the increasing demands for time slots in the curriculum would make it difficult for radiologists to give interactive and active lectures. Introducing more problem-based learning courses may improve overall class participation and even help provide an opportunity for radiologists to teach medical students without facing the issues mentioned earlier.^[35]^ Problem-based learnings require the students to actively research the topic surrounding a fictional patient and radiologists can answer and contribute to the lecture without facing the burdensome preparation that didactic lectures bring about.

## 5. Limitations

The present study has some limitations. First, the cross-sectional study enrolled relatively small samples using a convenience sampling technique. Second, this study mostly included students from the Sindh and Punjab provinces. Therefore, the results cannot be generalized to all medical students in Pakistan. The conduct of extensive studies from other provinces is essential to investigate the awareness and attitude of the students at the national level. Third, as a survey study, it depended on participants’ own subjective, rather than objective, views through self-assessment and self-reporting. Although this study was made available to all medical students, a response bias may be present toward the students who were potentially interested in considering radiology as a specialty. Finally, our study looks at students’ knowledge and attitudes regarding radiology at only one point in time; a longitudinal study would be a more accurate representation of medical students’ knowledge over time.

## 6. Conclusion

The results of this study indicate that medical students have suboptimal knowledge regarding the field of radiology, primarily due to insufficient exposure to radiology teaching in the undergraduate medical curriculum. Early specialty exposure plays a significant role in specialty selection. Hence, implementing and intensifying early student exposure of students to radiology within the undergraduate curriculum will encourage the growth of the specialty. Collaboration between radiological societies and medical schools to design an effective curriculum can promote development in the radiology field.

## Acknowledgments

Authors are grateful to Dr Osamah Alwalid (MBBS, MMed, FRCR) for reviewing the questionnaire of the study.

## Author contributions

**Conceptualization:** Muhammad Junaid Tahir, Hashaam Jamil

**Data curation:** Muhammad Junaid Tahir, Hashaam Jamil

**Investigation:** Razia Jabbar

**Methodology:** Razia Jabbar, Mohsin Khalid Qureshi

**Resources:** Mohsin Khalid Qureshi, Muhammad Hamayl Zeeshan

**Software:** Muhammad Hamayl Zeeshan

**Supervision:** Irfan Ullah

**Validation:** Irfan Ullah

**Visualization:** Abubakar Nazir

**Writing – original draft:** Abubakar Nazir

**Formal analysis:** Muna Malik

**Writing – review & editing:** Muna Malik, Mohammed Mahmmoud Fadelallah Eljack, Muhammad Sohaib Asghar

**Project administration:** Muhammad Sohaib Asghar
